# Supplementary material for: Complex‐centric proteome profiling by SEC‐SWATH‐MS
Source: Mol Syst Biol. 2019 Jan 14;15(1):e8438. doi: 10.15252/msb.20188438 (PMC6346213; doi:10.15252/msb.20188438)
Supplement: Supplementary file 8 — Dataset EV7 [file MSB-15-e8438-s008.zip › feature_plots_string/Q9NWU2.pdf]

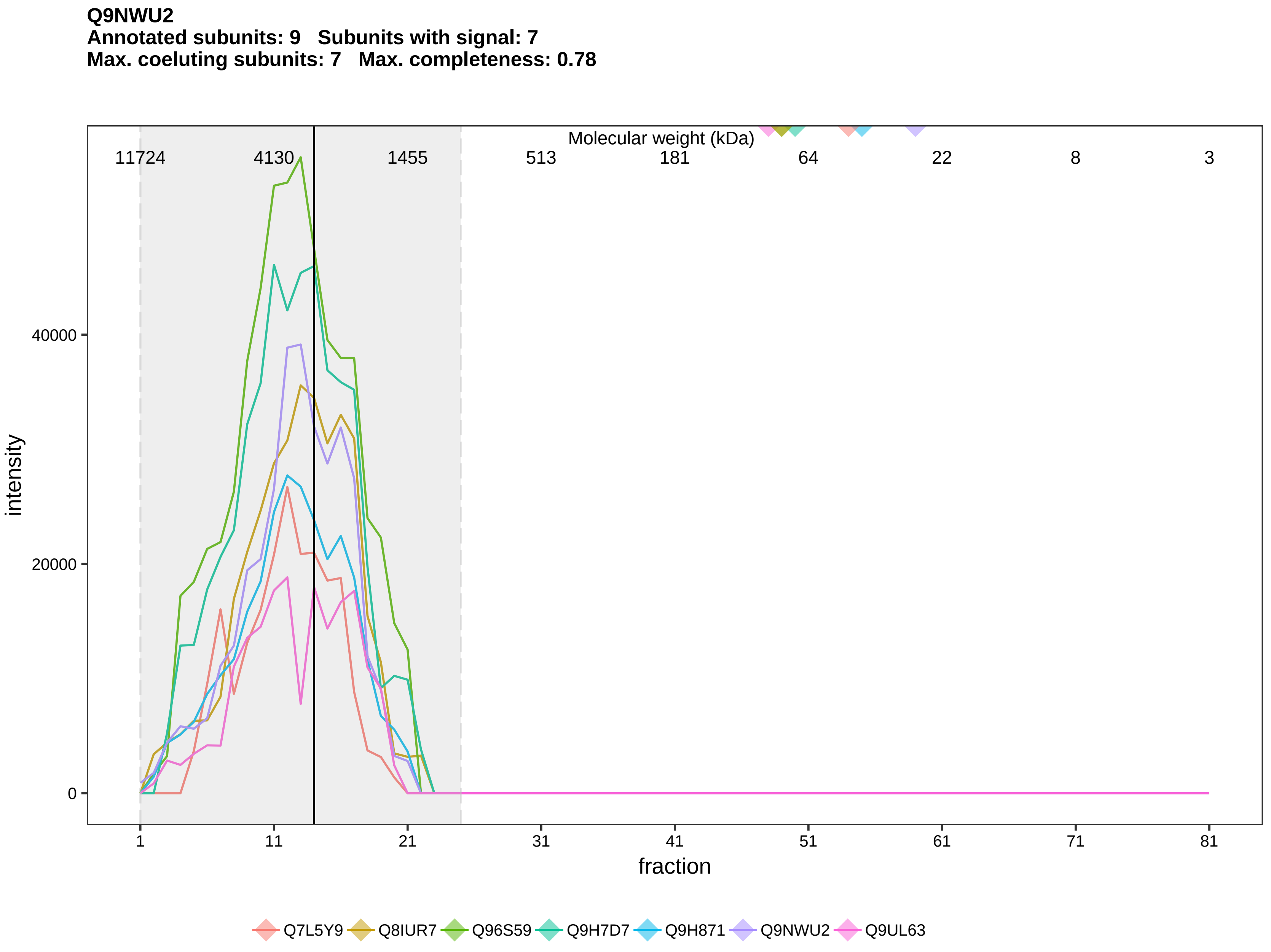
Q9NWU2
Annotated subunits: 9   Subunits with signal: 7
Max. coeluting subunits: 7   Max. completeness: 0.78
Molecular weight (kDa)
11724
4130
1455
513
181
64
22
8
3
intensity
40000
20000
0
1
11
21
31
41
51
61
71
81
fraction
Q7L5Y9
Q8IUR7
Q96S59
Q9H7D7
Q9H871
Q9NWU2
Q9UL63
